# Robust bioengineered 3D functional human intestinal epithelium

**DOI:** 10.1038/srep13708

**Published:** 2015-09-16

**Authors:** Ying Chen, Yinan Lin, Kimberly M. Davis, Qianrui Wang, Jelena Rnjak-Kovacina, Chunmei Li, Ralph R. Isberg, Carol A. Kumamoto, Joan Mecsas, David L. Kaplan

**Affiliations:** 1 Department of Biomedical Engineering, Tufts University, 4 Colby Street, Medford, MA 02155, USA; 2 Department of Molecular Biology and Microbiology, Tufts University School of Medicine, 145 Harrison Ave, Boston, MA 02111, USA; 3 Graduate School of Biomedical Engineering, UNSW Australia, Sydney, NSW, Australia

## Abstract

Intestinal functions are central to human physiology, health and disease. Options to study these functions with direct relevance to the human condition remain severely limited when using conventional cell cultures, microfluidic systems, organoids, animal surrogates or human studies. To replicate *in vitro* the tissue architecture and microenvironments of native intestine, we developed a 3D porous protein scaffolding system, containing a geometrically-engineered hollow lumen, with adaptability to both large and small intestines. These intestinal tissues demonstrated representative human responses by permitting continuous accumulation of mucous secretions on the epithelial surface, establishing low oxygen tension in the lumen, and interacting with gut-colonizing bacteria. The newly developed 3D intestine model enabled months-long sustained access to these intestinal functions *in vitro*, readily integrable with a multitude of different organ mimics and will therefore ensure a reliable *ex vivo* tissue system for studies in a broad context of human intestinal diseases and treatments.

Diseases associated with the human intestine, such as bacterial, viral and parasitic infections, irritable bowel syndrome and inflammatory bowel disease, have attracted considerable attention from biologists, microbiologists, immunologists and pharmacologists, as millions of people suffer from these maladies worldwide leading to significant gastrointestinal morbidity and mortality^1^. A lack of correlated human physiological responses renders living animals unsuitable models to study the causal factors and treatment cascades of human intestinal infections and disorders^2^,^3^. The current state-of-the-art in tissue engineering of human intestine *in vitro*, despite a promising alternative to animal models and an exceptionally powerful tool for *in-situ* visualization and continuous monitoring of the infectious processes^4^,^5^,^6^,^7^, has fallen short in demonstrating the elaborate tissue architecture and complex mucosal barrier functions of native intestines^8^.

*In vitro* formation of the double-layered mucus, of which the outer layer provides a habitat for commensal microbes and the inner layer dictates the first line of immune defense against invading pathogens, remains a particular challenge^9^. Mucus production has been readily achieved using mucus-secreting cells, but this implementation holistically involves a trade-off between functions of tight junctions and those of mucus^10^. Microfluidic-based mechanical stimulation methods, though proven capable of triggering increased mucus secretion from epithelial cells, are mainly addressed to the two-dimensional (2D) planar cell cultures^11^. Another pressing challenge for bioengineering models of human intestine lies in *in vitro* generation and dynamic control of an *in vivo*-like, oxygen-restricted luminal microenvironment^12^. 2D culture approaches to date, with limited geometrical tunability, variously rely on pre-gassed culture media^13^, and therefore negate the formation of natural oxygen gradients which are critical in driving functional outcomes, e.g. the reciprocal interaction between intestinal microbiota and altered epithelial responses^14^. Cystic organoids, while resembling the intestinal crypt-villus microtopography, develop fully enclosed luminal spaces and therefore hinder crosstalk between discrete mini-niches which thereby limits utility in studies of intestinal pathology, e.g. chronic infections that affect various sites along the gastrointestinal tract^4^. Regenerated intestine can be pursued by implanting intestinal organoids units into mice^15^,^16^, as more of an *in vivo* intestine model.

Here we report our progress towards the construction of a novel three-dimensional (3D) *in vitro* model of human intestine, using silk protein as a scaffolding material^17^,^18^,^19^,^20^, mimicking both the structure and function of native intestine. These modular intestine constructs feature a 3D geometrically-engineered hollow lumen lined with a monolayer of highly polarized epithelial cells, which are further supported and nourished by the surrounding myofibroblasts dispersed in the porous scaffold bulk. The resulting *in vitro* models properly represent the characteristic functions of human intestine, especially to maintain a continuous mucus double layer covering the epithelial surface and a progressive proximal-to-distal reduction in oxygen tension through the intestinal lumen. Sustained cultivation of the intestine system for months, together with the scalable and integrative nature of the tissue engineering techniques, could deepen understanding of human intestinal infection, disease and treatment.

## Results

### Compartmentalized human intestinal tissue on 3D silk protein scaffolds

**Figure 1:**
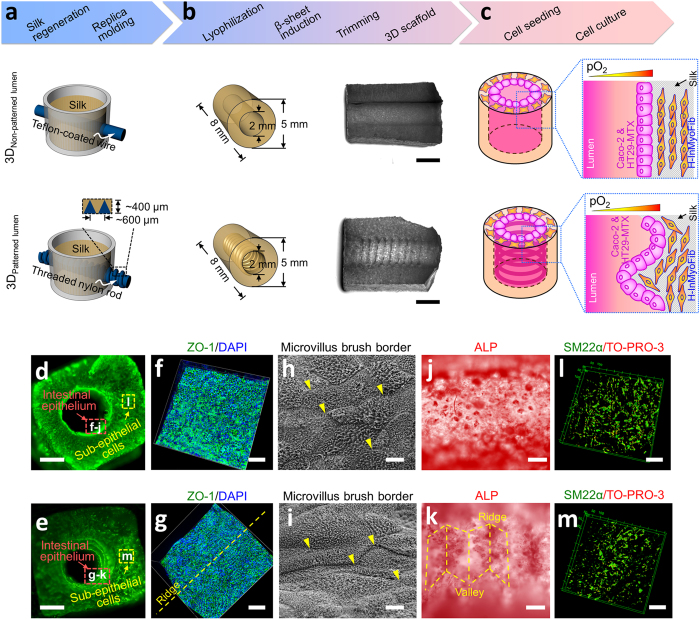
Human intestines on 3D porous silk scaffolds. (**a**,**b**) Schematics of the fabrication process for building silk-based porous scaffolds for 3D human intestine engineering. Silk scaffolds with hollow channels were prepared using a sequential six step process involving silk regeneration, cylindrical PDMS mold casting, insertion of Teflon-coated wires or Nylon screw across the cylinder, application of the silk solution, lyophilization and β-sheet induction. Upon completion of this process, the Teflon-coated wires or Nylon screw was removed, leaving a 3D, porous scaffold with a 2 mm diameter of non-patterned or screw-patterned (patterned, ridge like features with a height of 400 μm) hollow channels spanning the length of the scaffold and a bulk space that contained interconnected pores surrounding the channel. The scaffolds were then reproducibly trimmed along the axis of the hollow channels into 5 mm diameter × 8 mm long (mm) cylinders (b, Scale bars, 4 mm). (**c**) Schematics show the 3D non-patterned and patterned intestine system layout. (**d**,**e**) F-actin stain of 3D scaffolds without patterns (upper panel) and with patterns (lower panel) demonstrates how intestinal epithelial cells and myofiblasts localize in the 3D silk scaffolds. Scale bar, 1 mm. (**f–k**) 3D confocal images of ZO-1 immunostaining, SEM, and ALP staining on the epithelial cells seeded in the scaffold lumens demonstrated fully polarized epithelial cells. Scale bars, 100 μm (**f**,**g**), 1 μm (**h**,**i**), 200 μm (**j**,**k**). (**l**,**m**) 3D confocal view of immunostaining of SM22α on cells seeded in the bulk space (week 8) showed H-InMyoFibs keep their phenotype on the scaffolds. Scale bar, 50 μm.

Our approach to establish a porous silk scaffold system for intestine engineering and the cell seeding strategy are summarized in Fig. 1a–c. and the online methods. Smooth wires and threaded rods (screws) were employed during the scaffolding to generate non-patterned and patterned hollow channel compartments within the silk bulks to house the epithelial cells. We engineered intestine models by culturing human intestinal epithelial cells (Caco-2 and HT29-MTX cells) on the surface of silk scaffold lumens and primary human intestinal myofibroblasts (H-InMyoFibs) within the scaffold bulk space. Enterocyte-like Caco-2 and Goblet-like HT29-MTX cells are routinely used in the establishment of intestinal model systems for nutrient/drug absorption and host-pathogen interaction studies^21^, while H-InMyoFibs are fibroblasts that secrete cytokines and growth factors to support the growth, differentiation, and expansion of the human intestinal epithelium^22^. Caco-2 and HT29-MTX cells (3:1, Supporting Information Fig. 1, a ratio reflects the native intestine population^23^) were injected into the hollow channels allowing the luminal attachment and growth. The primary human intestinal myofibroblasts (H-InMyoFibs) were delivered into the porous scaffold bulk space to support the growth of the epithelial cells. After cell seeding, we first performed F-actin staining on the scaffolds to visualize the general spatial distribution of cells in the engineered intestinal tissues at day 10 post cell seeding (Fig. 1d,e) and then used specific biomarkers to discriminate between the epithelial cells and H-InMyoFibs seeded on the different compartments of the scaffolds (lumen lining vs bulk sponge, Fig. 1f–m). The staining results using cell-specific biomarkers indicated that the design of the scaffolds (non-patterned or patterned lumens) and cell seeding strategy (lumen lining epithelial cells vs. bulk sponge seeded H-InMyoFibs) successfully localized different cell lineages within the scaffold compartments as planned; intestinal epithelial markers (ZO-1, E-caherin, and villin) were detected in the hollow channel surface (Fig. 1f,g and Supporting Information Fig. 2) and a myofibroblast marker (SM22α) was detected in the scaffold bulk (Fig. 1l,m). Cells grown on scaffolds formed columnar structure (Supporting Information, Fig. 3). Fully polarized epithelial cells were also demonstrated by the high packing density of microvilli with continuous brush borders across the cells (Fig. 1h,i and Supporting Information Fig. 4), and the apical alkaline phosphatase (ALP) enzyme activity (Fig. 1j,k). H-InMyoFibs in the scaffold bulk were processed for Calcein-AM staining (live cell staining) to determine cell seeding efficiency and proliferation in the bulk (Supporting Information Fig. 5). The H-InMyoFibs were evenly dispersed and began to spread in the bulk space after 24 hours (Supporting Information Fig. 5a). The pores of the scaffolds supported H-InMyoFibs for at least 8 weeks (Supporting Information Fig. 5b–d), and the deletion of H-InMyoFibs in the co-culture systems resulted in shorter-term growth and differentiation of the epithelial cells based on the analysis of scaffolds containing only the Caco-2/HT29-MTX cells (Supporting Information, Fig. 6).

### Mucus layer production

**Figure 2:**
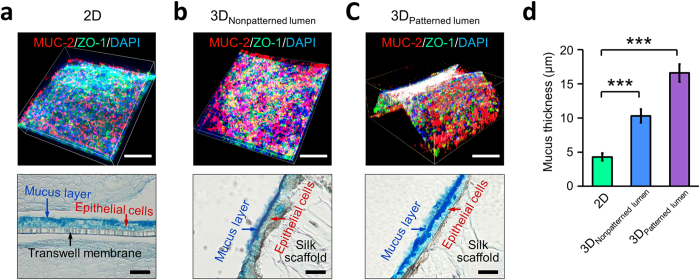
3D silk scaffolds increase the thickness of the mucus layer of the intestinal epithelial cells. (**a–c**) Confocal microscopy images of immunostaining of MUC-2, ZO-1, and DAPI of Caco-2/HT29-MTX cultured on 2D transwells (**a**), and non-patterned (**b**) and patterned (**c**) 3D silk scaffolds 21 days post cell seeding. MUC2 is visualized as red, ZO-1 as green, and DAPI as blue. Scale bar = 200 μm. (**d,e**) Light microscopy of toluidine blue stained frozen sections across the transwell inserts (**d**), non-patterned (**e**) and patterned (**f**) 3D silk scaffolds. Outer mucus layers on 3D scaffolds are pointed by the black arrowheads, while inner mucus layers are pointed by yellow arrowheads. Scale bar = 100 μm. (**g**) Measurement of mucus thickness yielded by the epithelium grown on 2D and 3D systems. n = 6 in each group, ***p < 0.001.

Next, we studied mucus secretion by dissecting the mucus layers. Epithelial cells grown on 2D transwells with the same initial number of cell seeding and the support of H-InMyoFibs were used as controls. We carried out our analyses of the thickness of mucus layer on day 21 post seeding, as mucus production identified in both transwell and 3D scaffold systems increased from day 10, reaching a peak on day 21. We first detected the expression of MUC2, a structural component of the intestinal mucus layer, by immunostaining to localize the mucus layers. At day 21, full coverage of MUC2 on the epithelia with tight junctions was found in co-cultures on both transwell inserts and scaffold lumens. The 3D constructs showed an increase in luminal MUC2 fluorescence intensity compared to the transwells (Fig. 2a–c). Next, Alcian blue staining of cross-sections of these constructs was used to measure the thickness of the mucus layers. The intestinal epithelial cells on the 3D scaffolds 21 days post seeding resulted in a cell monolayer coated with a compact inner layer and a loose outer layer of mucus (Fig. 2e,f). The mucus layers were approximately 11 and 17 μm thick in the 3D non-patterned and patterned scaffolds, respectively (Fig. 2g). In contrast the mucus layer was only about 4 μm thick in the 2D system without a clear distinction between the inner and outer layers (Fig. 2d).

### Intraluminal oxygen gradients

**Figure 3:**
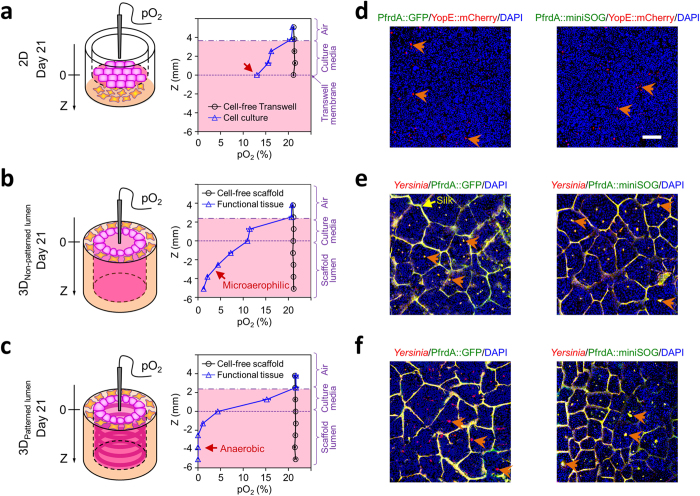
3D bioengineered human intestinal tissues mimic *in vivo* luminal oxygen levels. (**a**) Confluent intestinal epithelial monolayers cultured on transwell inserts under the aerobic condition. (**b**) Concentration profile of oxygen at different depths down the lumen of 3D tissues (with plain luminal surfaces) demonstrated ready access to microaerobic and nanaerobic oxygen concentrations. (**c**) The increased epithelial area in 3D tissues achieved by patterning the luminal surfaces further extended the low end of the oxygen profile to below the anaerobic threshold. All measurements were performed on day 21 post-confluence. (**d**) Red fluorescence of the *yopE* reporter (arrowheads) on transwell inserts, in the absence of *frdA*-driven green fluorescence (GFP or miniSOG), verified that bacteria experience aerobic conditions. (**e**) Yellow fluorescence (arrowheads) (mixed color of red anti-*Yersinia* and green GFP or miniSOG) in non-patterned lumens indicates bacteria experience microaerobic conditions. (**f**) Red *Yersinia*, detected by antisera, only expressed miniSOG (arrowheads), revealing bacteria experience strictly anaerobic conditions in patterned lumens. Scale bar = 50 μm.

A major advantage of our 3D scaffold system is the hollow channel compartment to house the epithelial cells. To explore if the cells in the 3D lumen would generate a different oxygen profile in the lumen than the cells grown on the open, exterior growth surface in transwells, an oxygen probe was used to quantify oxygen levels (Fig. 3). In the 2D cultures on transwell inserts (Fig. 3a), an oxygen tension of 13% was maintained with the confluent epithelial monolayer. In contrast, the intestinal epithelial cells lining the interior surfaces of the 3D scaffolds experienced decreased oxygen levels that mimicked *in vivo* conditions. Oxygen diffusion into the cell cultures, driven by this vertical oxygen gradient was dictated by the oxygen consumption kinetics and metabolic activities of the cells, based on cell-free transwell controls. The 3D models of human intestinal tissue (Fig. 3b,c) exhibited depth-graded oxygen profiles in the luminal direction. In Fig. 3b, a region of microaerobic conditions (pO_2_ between 5% and 2%) was identified at depths ranging from 2 to 5 mm into the non-patterned intestinal lumen, bordered by a nanaerobic region (pO_2_ ~ 1%) at the depth of 5 to 6 mm. Patterned scaffolds and the resulting intestinal lumens (Fig. 3c) added a new dimension to the oxygen profiles. A highly oxygen-deficient, anaerobic condition (pO_2_ < 0.1%) was found at a depth of 2 to 6 mm in the patterned lumens. Interestingly, these proximal-to-distal gradients *in vitro* corresponded to the presence of a marked decrease in the luminal oxygen tension along the gastrointestinal tract, e.g. 3 ~ 7% in the mid-stomach, 2 ~ 4% in the mid-duodenum, ~1% in the mid-small intestine, and <0.4% in the distal colon in a living mouse^24^.

### Interaction with engineered *Yersinia* oxygen-sensing fluorescent reporters

To confirm the low oxygen tensions measured above in the lumen of the 3D intestinal tissues, we performed *in situ* detection of oxygen levels in different culture systems by using engineered oxygen-sensing fluorescent reporter strains of the bacterium *Yersinia pseudotuberculosis. Y. pseudotuberculosis* is an enteric bacterial pathogen that colonizes the human intestinal tract through direct interaction with the intestinal epithelium^25^. Infection with this bacterium should help determine whether the 3D system can support a physiological infection scenario. Additionally, because this bacterium is capable of growth under aerobic and anaerobic conditions, we can utilize fluorescent reporter strains to detect the presence or absence of oxygen. The promoter of the hypoxia-responsive respiratory enzyme, fumarate reductase, was used to drive expression of the fluorescent protein genes, *gfp* or mini singlet-oxygen generator (*miniSOG)*^26^,^27^. The GFP-tagged fusion protein folds in an oxygen-dependent manner, while the mini-SOG tag folds in an oxygen-independent manner (Supporting Information Fig. 7). MiniSOG expression (*P*_*frdA*_*::miniSOG*) was detected exclusively in *Y. pseudotuberculosis* colonizing the 3D luminal epithelia (Fig. 3e,f, right), confirming the observed low oxygen tension measurements within 3D scaffolds. In contrast, on the 2D epithelial cell layer, neither expression of *P*_*frdA*_*::gfp* nor *P*_*frdA*_*::miniSOG* was detected, although mCherry expression was visualized in clusters of *Y. pseudotuberculosis* (Fig. 3d), driven by the temperature-inducible, *Yersinia* type-III secretion system effector protein *yopE (yopE::mCherry)*^28^,^29^. These results confirmed that the 2D epithelial cell layers represent aerobic culture conditions, based on a lack of *frdA*-driven signals, and the presence of mCherry fluorescence, which requires oxygen to fold (Supporting Information Fig. 7). Furthermore, the comparison of miniSOG and GFP fluorescent intensities expressed in the *Y. pseudotuberculosis P*_*frdA*_*::miniSOG* and *P*_*frdA*_*::gfp* colonies was used to identify physiological threshold levels of oxygen concentration in the 3D intestinal lumens^30^. The presence of GFP fluorescence in the *Y. pseudotuberculosis* colonizing the non-patterned epithelium (Fig. 3e, left), and its absence when colonizing the patterned counterpart (Fig. 3f, left), clearly distinguished between the microaerobic conditions of the non-patterned scaffolds and the anaerobic conditions of the patterned scaffolds (Supporting Information Fig. 7). Finally, the interaction of the 3D intestinal tissues with the bacterial strains potentially supports the utility of this 3D system to study bacterial interactions/colonization, such as those related mechanisms of infection, impact of normal microbiota and related questions.

### Long term cultures of intestinal epithelium

**Figure 4:**
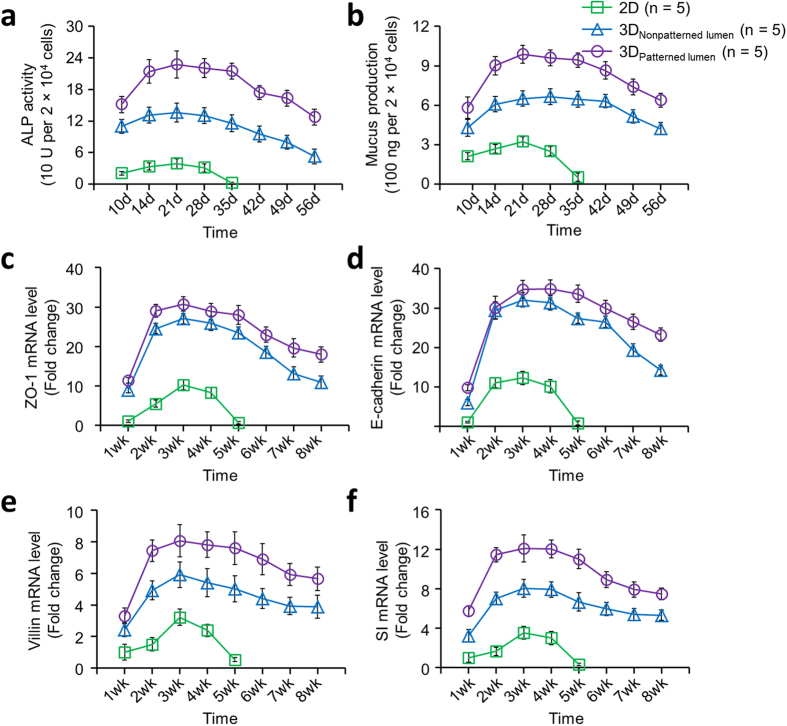
3D architecture and multicellular co-culture system significantly enhanced the overall differentiation level of the epithelial cells and supported longer-term static cultures of intestinal epithelium. (**a**,**b**) Quantification of alkaline phosphatase activity (**a**) and mucus production (**b**) of intestinal epithelial cells cultured on 2D transwell and 3D silk scaffolds. (**c–f**) Gene expression levels of cell junction-related genes and intestinal epithelial biomarkers, including ZO-1 (**c**), E-cadherin (**d**), Villin (**e**), and SI (**f**), were evaluated by quantitative reverse transcription-polymerase chain reaction (qRT-PCR). Data is presented as mean ± SEM, n = 5 in each group, p < 0.001.

To investigate whether the cultivation of intestinal epithelium in 3D structured scaffolds with co-cultures of H-InMyoFibs promoted long-term maintenance of its tissue function and cell phenotype^31^, the production of ALP and mucus, and epithelial differentiation-related gene expression (ZO-1, E-cadherin, Villin, sucrase-isomaltase (SI)) from the epithelium grown in 3D constructs over time were quantified. Generally, cell cultures on the 3D scaffolds were maintained for longer time frames (up to at least 8 weeks) than on transwell inserts (Fig. 4). After about 4 weeks the cultures on transwells started to disintegrate and detach due to cell overgrowth and multilayer formation. In contrast, co-cultures grown on 3D scaffolds were intact at the 8 week time point (Supporting Information, Fig. 8). In addition, the overall expression levels of all markers from cells on 3D scaffolds were significantly higher than on transwells across all time points; patterned scaffolds generated relatively higher levels of all markers when compared to the non-patterned 3D scaffolds. There was no significant difference between mRNA levels for ZO-1 and E-cadherin in the patterned and non-patterned groups (Fig. 4c,d). These data demonstrated that the 3D silk scaffolding systems improved cell function for extended time frames with higher expression of all intestinal markers. These results suggest a functional and more physiologically-relevant intestinal epithelium system generated in the 3D designs which also provides critical sustainable systems for the study of chronic impacts of diseases, drugs and microbiota *in vitro* in future work with these systems.

## Discussion

Our bioengineered 3D human intestine model using silk protein-based scaffold demonstrated its powerful and enduring strength to reconstitute and sustain both architectural and functional imperatives of human intestine *in vitro*. All steps and processes of intestinal tissue modeling can be scaled and automated related to the system designs. This robust protein scaffolding method differs substantially from existing *in vitro* approaches in its profound reflection of the 3D compartmentalized structure and complex multicellular organization of the native tissue^9^. Physical characteristics of different intestine regions, including lumen dimension, shape and surface features, can be instantiated to improve the model’s site specificity and biological relevance. On the other hand, cellular diversity of the 3D culture can be further expanded, e.g. to encode a hierarchy of innate immune responses, by using different cell configurations (Supporting Information text 1 and 2).

The construction of a central hollow lumen, with emphasis on its tunable surface topography, enabled us to reconstruct a 3D-supported, intestinal epithelial interface *in vitro*. For instance, these 3D intestine constructs exhibited continuous mucus accumulation, with thickness ranging between 10 and 20 μm, which constitutes an indispensable prerequisite for *in vitro* studies of human intestinal host-microbial interplay^32^. In addition, this mucus-protected 3D epithelium monolayer, with the apical surface facing inward towards the lumen space and the basal surface associated with underlying myofibroblasts, autonomously established a microenvironmental gradient of oxygen tension, decreasing from proximal to distal regions in an *in vivo*-like fashion. This bioengineered 3D system, therefore, facilitated ready access to the full range of luminal oxygen conditions *in vivo*, under either physiological stresses or pathological stimuli. The depth-graded oxygen profiles reflect a kinetic limitation in oxygen transport as a result of the competition between the oxygen consumption of cells and the diffusion of oxygen into culture media. The dramatically lower pO_2_ in the 3D patterned system may be due to the increased epithelial cell mass per unit of length of the lumen. At the high-oxygen end of the spectrum, for example, microaerophilic conditions can be created and controlled in the surface-engineered, small intestinal lumen by dynamically perfusing the scaffolds to enhance the oxygen supply to the villus epithelial surfaces. Instead, at the fully anaerobic end, 3D tissue scaffolds with plain lumens could be cultured in a hypoxic chamber or the luminal channel can be perfused with culture media pre-equilibrated with strict anaerobic conditions.

More closely representing a multitude of *in vivo* human intestinal responses, our 3D intestine tissue constructs have been shown to potentially support the *in vitro* luminal colonization of intestinal bacteria, highlighting the potential to recapitulate *in vivo* effects of the gut microbiota dominated by anaerobic bacteria^12^. This was demonstrated when using wild-type pathogenic strains of *Yersinia* as oxygen level indicators, as well as by the co-culture of a probiotic strain of *Lactobacillus rhamnosus* GG with epithelial cells in the lumens (Supporting Information Fig. 9).

Long-term viability of this bioengineered 3D intestine system offers particular benefits for *in vitro* studies of chronic or recurrent intestinal infections, when multiple sites along the gastrointestinal tract are usually involved and the treatment normally takes a relatively long period. One limitation of this system is that the tissue functions tend to decrease after a few weeks post cell seeding; perfusion of these 3D scaffolds by a bioreactor system may help to achieve dynamic flow and enhance intestinal functional performance over longer time frames. We envision that the new 3D intestine model presented here, readily integrable into a series of human organ mimics, will shed new light on the interconnected and interdependent roles of intestinal functions in a broad context of human well-being.

## Methods

### Cell lines

The Caco-2 (CRL-2102) cell line was obtained from ATCC (Rockville, MD), and HT29-MTX cell line was obtained from the Publich Health England Culture Collections (Salisbury, Great Britain). Both Caco-2 and HT29-MTX cells were grown in DMEM supplemented with 10% fetal bovine serum, 10 μg/mL human transferrin (Gibco), and 1% antibiotics and antimycotics. In addition, human Intestinal Myofibroblasts (H-InMyoFib) were purchased from Lonza and cultured in SMGM™-2 BulletKit™ medium (Lonza). Cells were cultured in T-175 cm^2^ tissue culture flasks (Corning), maintained at 37 °C, 5% CO_2_ humidied atmosphere and harvested with 0.25% trypsin-EDTA (Gibco) prior to seeding. For Caco-2 and HT29-MTX, cells from passage number 38–50 were used for the experiments. For H-InmyoFib, passage 3–5 was used.

### 3D silk fibroin protein scaffold fabrication

#### Silk Solution Preparation

Silk fibroin was extracted from *Bombyx mori* silk worm cocoons. 5 g of cocoons were cut into small pieces and boiled for 30 min in 2L of 0.02 M Na2CO3 solution. The degummed fibers were then rinsed thoroughly with deionized water to remove residual Na2CO3 solution and air dried overnight. The dried silk fibers were dissolved in 9.3 M LiBr at 60 °C for 4 h. Then the fibroin solution was dialyzed against deionized water for 3 days to yield fibroin water solution. The final concentration of the aqueous silk fibroin solution was about 5–6 wt%, which was determined by air drying a known volume of silk solution and massing the remaining solids. Silk solution was diluted to 4 wt% with deionized water for use.

#### Preparation of polydimethylsiloxane (PDMS) molds

To prepare silk scaffolds with hollow channels in them, special cylindrical molds were cast from olydimethylsiloxane (Down Corning). PDMS was prepared by mixing the base reagent with the curing reagent in a mass ratio of 10:1. The PDMS was cured at 60 °C for 2 hours and then delaminated from the plates. The cylindrical PDMS molds (Fig. 1e) consist of a Teflon-coated stainless steel wire (Ø = 2 mm, McMaster-Carr) or a Nylon pan head machine screw (Slotted, 4–40 Thread, 1” Length, McMaster-Carr) inserted through the cross-section of the cylinder to develop a hollow channel without pattern or with a screw pattern in the silk scaffold.

#### Fabrication of porous 3D silk scaffolds by freeze drying

4–5 wt% viscous silk solution was poured into the PDMS molds. The molds were frozen at −20 °C overnight and then transferred to a lyophilizer for silk drying. Dried silk scaffolds were then autoclaved to induce a β-sheet conformation, soaked in distilled water overnight, and trimmed along the axis of the hollow channel into a 5 × 5 × 8 (mm) cuboid. The fabrication method resulted in a scaffold consisting of a non-patterned or screw patterned (patterned) hollow channel space (2 mm in diameter) and a bulk space around the channel that contained interconnected pores (Fig. 1f,g). The 4–5 wt% silk solution has been used previously to generate scaffolds with 150–250 μm interconnected pores useful for cell attachment and growth^17^,^33^.

### Co-culture of Caco-2, HT29-MTX, and H-MyoFib in 2D transwell system

H-MyoFib cells were plated on the bottom of the 24-well transwell cell culture system (Pore size 0.4 μm; Costar Corp.) using the complete media and culture environment as described above. The Caco-2 and HT29-MTX cells were cultured on the membrane of transwell cell culture inserts at a density of 2 × 10^5^/cm^2^ and allowed to attach for 2 hours using the conditions above. Caco-2/HT29-MTX cells cultured on membrane transwell inserts were placed into the 24-well plates containing the H-MyoFib to initiate the experiment. Cells co-cultured in transwells were maintained routinely in DMEM: SMGM (1:1) up to 40 days.

### Cell seeding on 3D silk scaffolds

The hollow channel of the 3D scaffolds was used to accommodate human intestinal epithelial cells (Caco-2 and HT29-MTX cells), while the porous bulk space was used house primary human intestinal myofibroblasts (H-InMyoFibs). Collagen gel was used to deliver H-InMyoFibs into the spongy silk scaffolds^34^. Collagen gels containing 2 × 10^5^ H-InMyoFib per ml were prepared. Gels were composed of a 10% cell suspension of H-InMyoFib (in 10% 1xDMEM (Gibco), 10% 10xDMEM (Sigma-Aldrich), and 80% type I rat tail collagen 2.01 mg/ml; (First Link, UK)). Gels containing H-InMyoFib were then delivered into the spongy silk scaffolds with the Teflon-coated stainless steel wires or Nylon pan head machine screws on to leave the non-patterned and screw-patterned hollow channel open for the seeding of Caco-2 and HT29-MTX. After 20–25 minutes of gelation at 37 °C, the Teflon-coated stainless steel wires or Nylon pan head machine screws were carefully removed from the scaffolds. After that, the non-patterned and patterned hollow channels were loaded with the Caco-2/HT29-MTX cells (3:1) at a density of 4 × 10^6^ cells/mL (Supporting Information Fig. 1). Afterwards, scaffolds loaded with the Caco-2/HT29-MTX cell suspension were incubated at 37 °C for 2 hours, and then were flipped down and incubated for another 2 hours. During the incubation, a small amount of SMGM medium was dripped on the scaffolds to keep them moistened.

### Immunofluorescence and confocal imaging

Transwells and silk scaffolds with intestinal cells were fixed with 4% paraformaldehyde (PFA, Santa Cruz). Silk scaffolds were cut in half along the longitudinal axis in order to better expose the lumen to the blocking solutions and antibodies during the following incubation steps. All specimens were then permeabilized using 0.1% Triton x-100 in phosphate-buffered saline (PBS, Gibco), then blocked with 5% bovine serum albumin (BSA, Sigma) for 2 hours. These specimens were incubated overnight at 4 °C with anti-human ZO-1(BD Transduction Laboratorie, 1:50), anti-e-cadherin (abcam, 1:50), anti-human-MUC-2 (Santa Cruz Biotech, 1:50) and anti-Villin (abcam, 1:100), then immersed in Alexa Fluor 488 donkey anti-mouse and Alexa Fluor 546 goat-anti-rabbit secondary antibodies (Invitrogen) at a dilution of 1:100, respectively. Scaffolds were then counterstained with dihydrochloride (DAPI; Invitrogen) before being mounted using Vectashield mounting medium (Vector Laboratories). For live staining, calcein-AM (Invitrogen) was used at different time points, following manufacturer’s guidelines. These 3D scaffolds were scanned using a Leica SP2 confocal microscope (Leica Microsystems) and Nikon A1R (Nikon Instruments Inc.) with Z-series capability. Scaffolds were observed under a confocal microscope with a filter set for DAPI (Ex/Em: 350/470 nm), Texas Red (Ex/Em: 540/605 nm) and GFP/FITC (Ex/Em: 488/514 nm). 3D rendering images and confocal 3D maximum projection images were assembled with Leica confocal software (ver 2.61, Leica), NIS-Elements AR software package (ver 4.20.01, Nikon) and ImageJ.

### Alkaline phosphatase (ALP) stain and assay

ALP staining^15^ was performed using the Vector Red Alkaline Phosphatase Substrate Kit I (VECTOR Laboratories), according to the manufacturer’s protocol. Briefly, transwells and silk scaffolds with cells were fixed with 4% paraformaldehyde for 1 minute at room temperature, then washed two times with PBS. The specimens were incubated with substrate solution at room temperature until suitable staining developed, then were imaged with the Olympus mvx10 macroscope and captured by cellSens Dimension (ver 1.8.1) program. ALP activity generated from intestinal epithelial cells grown on each system was quantified using a protocol adapted from a previous study^35^. Generally, after the removal of culture medium, the transwell inserts and scaffolds were washed twice with PBS and incubated with 200 μl of p-Nitrophenyl phosphate prepared from Sigma fast p-Nitrophenyl phosphate tablets (Sigma) for 30 minutes at 37 °C. The plates containing the specimens were shaken every 10 minutes. After the incubation period, plates were moved onto ice, and the yellow soluble end product (p-Nitrophenol, p-NP) of the enzymatic reaction was harvested in 96-well-plates and the absorbance was immediately read at 405 nm using a Spectramax M2 Multimode Microplate Reader (Molecular Devices). The absorbance readings were compared to a standard curve to determine the concentration of the pNP that evolved. The ALP enzyme activity results were expressed as U = nmol of p-NP/min/20,000 cells.

### Mucus detection

For detection of mucus thickness, specimens were fixed in 4% PFA-1% GA, suspended in 20% sucrose overnight at 4 °C, mounted in OCT compound (Sakura Finetek), and cryosectioned at 12 μm. Sections were stained with 0.25% Alcian blue as described^36^. The staining process was as follows: the slides were dried in the air for 20 minutes; after rinsing in 3% acetic acid (Sigma) for 2 min, the slides were stained for 15 minutes in 0.25% Alcian blue 8GX (Sigma) in 3% acetic acid (pH 2.5); removed and dipped in 3% acetic acid and rinsed in running tap water for 10 minutes; the slides were dehydrated through an alcohol series, cleared in xylenes, and coverslipped with DPX (Electron Microscopy Sciences). Stained specimens and slides were evaluated by light microscopy. For the measurement of mucus thickness on slide samples, a minimum of ten different measurements were made perpendicular to the inner mucus layer per field. The measure fields in the 3D patterned lumen were randomly selected, including fields from both ridges and valleys. Twenty randomly selected fields were analyzed for each transwell or scaffold sample by using Image J with Fiji plugin. Quantification of mucus secretion of cells grown on tranwell inserts and silk scaffolds was determined by Enzyme-linked Lectin Assay (ELLA). The mucus layer was removed from the cell surface by washing transwell inserts or scaffold lumens by agitation on a shaker 135 rpm for 10 minutes with 1 mL FBS-free DMEM. The media were collected and the procedure was repeated twice. Toluidine blue staining on the samples being washed verified that the samples were mucus-free after the washing procedure. For each 5 specimens, the collected media containing mucus was centrifuged at 4,200 rpm for 15 minutes, diluted in sodium carbonate buffer (0.5 M, pH 9), added into 96 well microtiter plates, and incubated overnight at 4 °C. The wells were then washed with 0.05% Tween 20 in PBS (PBST), and blocked with 0.5% BSA in PBS. After washing with PBST, wells were incubated with 100 μL WGA-peroxidase (5 μg/mL) for 1 hour at 37 °C. The color reaction was obtained by adding 100 μL O-phenylenediamine (Sigma) to each well and keeping the wells at room temperature for 10 minutes. The reaction was stopped by adding 25 μl 3 M sulfuric acid to each well. The absorbance was read at 492 nm using a microtiter plate reader and converted to concentration using standard curve of porcine mucin in sodium carbonate buffer (0.5 M). The ELLA results were normalized to nanogram of mucin per 20,000 cells.

### Scanning Electron Microscopy (SEM) and quantification of microvilli

Transwells and silk scaffolds with cells were cross-linked with 2.5% GA, followed by progressive dehydration in a graded series of ethanols (30%, 50%, 75%, 95% and twice in 100%, 30 minutes at each concentration). The samples were subsequently dried by critical point drying with a liquid CO_2_ dryer (AutoSamdri-815, Tousimis Research Corp.). Prior to imaging using a scanning electron microscope (Zeiss UltraPlus SEM or Zeiss Supra 55 VP SEM, Carl Zeiss SMT Inc.) at a voltage of 2 ~ 3 kV, the samples were coated with a thin layer (10 nm thick) of Pt/Pd using a sputter coater (208HR, Cressington Scientific Instruments Inc., Cranberry Twp). For the quantification of microvilli, 100 × 1 μm^2^ of the cell surface area on each sample were randomly selected for the quantification of total number of microvilli using Image J. Ten samples were assessed for each group.

### Measurements of oxygen profiles *in vitro*

The oxygen concentration profiles were measured using a PC-controlled Microx TX3 oxygen meter (PreSens Precision Sensing GmbH, Rengensburg, Germany) equipped with a needle-type housing fiber-optic oxygen sensor (NTH-PSt1-L5-TF-NS40/0.8-OIW, 140 μm fiber tapered to a 50 μm tip). Prior to use, a two-point calibration was performed according to the manufacturer’s protocols with oxygen-free water (1% sodium sulfite, Sigma) corresponding to the 0% oxygen partial pressure and with air-saturated water corresponding to 100%. The needle probe was mounted on a custom-made micromanipulator^37^ capable of precisely positioning the measurement spot in the vertical direction. One complete turn of the screw knob resulted in 0.1 inch (2.5 mm) of travel. The human intestinal cells were cultured in 2D and 3D structures for 21 days post seeding. Each of the 3D intestinal tissue scaffolds was then placed in an Eppendorf tube with its luminal direction oriented perpendicularly, and allowed to stabilize for 1 to 2 hours before taking measurements. In each step of probe advancement (0.05 inch/step), the oxygen tension reading was allowed to equilibrate for at least 5 minutes followed by data recording. At the end of each depth-profile measurement, the probe was retracted and the process was repeated 3 times for each sample. Five oxygen readings (30 sec/reading) were collected at each measurement position, subsequently averaged and plotted (Fig. 3). To ensure the comparability between different samples, all three profiles were determined on the same day (within 6 hours) using the same probe and calibration.

### Bacterial strains & growth conditions

The wild typte (WT) *Y. pseudotuberculosis* (Yptb) strain, YPIII, was used for binding experiments. Bacteria were grown overnight into stationary phase in 2xYT broth (LB, with 2x yeast extract and tryptone) at 26 °C with rotation, and 4 × 10^6^ total CFUs were added to each scaffold, and 3.5 × 10^7^ total CFUs were added to transwells.

The probiotic *Lactobacillus rhamnosus GG* (LGG) ATCC 53103 strain was inoculated into MRS broth (de Man-Rogosa-Sharpe, Oxoid) and incubated at 37 °C in a humidified 5% CO_2_ incubator. The bacterial cells were harvested at the mid-log phase of growth (O.D.600 = 0.6) by centrifugation (3,000 × g, 10 min, 4 °C), washed with PBS and resuspended to an O.D.600 of 0.1 (~10^7^ cells/mL)^38^ in a mixture of MRS and antibiotic-free DMEM (1:1, v/v).

### Generation of reporter constructs

The WT *Y. pseudotuberculosis yopE::mCherry* strain has been previously described^29^, and contains *mCherry* cloned immediately downstream of *yopE*. Transcriptional fusions were constructed by fusing the *frdA* promoter region to *gfp* or *miniSOG*^39^, using overlap extension PCR. Transcriptional fusions were cloned into the low copy plasmid, pACYC184. qRT-PCR was used to confirm reporter expression (*gfp* or *miniSOG*) reflects *frdA* gene expression under different culture conditions.

### Bacterial binding experiments and co-culture of LGG on 3D silk scaffolds

Bacterial binding experiments and co-culture of LGG were performed on intestinal tissues at day 20–21 after cell seeding. Prior to bacterial inoculation, monolayers on transwells and scaffolds were washed with PBS, and cultured with fresh antibiotic-free medium supplemented with 5% deactivated FBS for 24 hours. For bacterial binding experiments, cells on transwell inserts were infected with Yptb strains containing either *P*_*frdA*_*::miniSOG* and *yopE::mCherry* or *P*_*frdA*_*::gfp* and *yopE::mCherry* fluorescent reporter constructs, while cells on scaffolds were infected with Yptb containing either *P*_*frdA*_*::miniSOG* or *P*_*frdA*_*::gfp*. For co-culture of LGG, the *Lactobacilli* were inoculated onto both 2D- and 3D-grown epithelial monolayers (at a bacteria to epithelial cell ratio of ~10:1). Bacterial cells were allowed to bind the intestinal epithelium for 30 minutes, and then non-adherent bacteria were removed, followed by 2 hours incubation for bacterial binding experiments and 4 hours for LGG co-culture at 37 °C in 21% CO_2_. Cells were washed and fixed for imaging. Total bound Yptb were detected within scaffolds using *Y. pseudotuberculosis*-specific rabbit antisera with a goat anti-rabbit Texas Red-conjugated secondary antibody (Molecular Probes). Constructs with LGG were stained with BacLight™ Red Bacterial Stain (Invitrogen) and DAPI. These specimens were then scanned by confocal microscopy as mentioned above.

### Quantitative RT-PCR

Intestinal epithelial cells on the transwell inserts or on the luminal surface of scaffolds were detached with 0.25% trypsin-EDTA. Total RNA was isolated using the Qiagen Mini mRNA Extraction kit. RNA was reverse-transcribed using High-Capacity cDNA Reverse Transcription Kit (Invitrogen) following the manufacturer’s instructions. Six nanograms of cDNA were used for real-time PCR amplification for each well, using primer sequences shown in table S1. For each gene tested we performed three experimental replicates and four biological replicates. Gene expression levels were normalized to the GAPDH mRNA level.

### Statistical Analysis

Data are presented as mean ± SEM (*n* = 5–10). A two tailed t-test was performed to compare means between two groups, and Analysis of Variance (ANOVA) was performed to compare means of multiple groups. P-values ≤ 0.05 were considered significant.

## Additional Information

**How to cite this article**: Chen, Y. *et al.* Robust bioengineered 3D functional human intestinal epithelium. *Sci. Rep.* **5**, 13708; doi: 10.1038/srep13708 (2015).

## Supplementary Material

Supplementary Information

## References

[b1] Park K. T. & Bass D. Inflammatory bowel disease-attributable costs and cost-effective strategies in the United States: a review. Inflamm Bowel Dis 17, 1603–1609 (2011). 21053357 10.1002/ibd.21488

[b2] Chang C. & Miller J. F. Campylobacter jejuni colonization of mice with limited enteric flora. Infect Immun 74, 5261–5271 (2006). 16926420 10.1128/IAI.01094-05 PMC1594848

[b3] Havelaar A. H. *et al.* Immunity to Campylobacter: its role in risk assessment and epidemiology. Crit Rev Microbiol 35, 1–22 (2009). 19514906 10.1080/10408410802636017

[b4] Sato T. *et al.* Single Lgr5 stem cells build crypt-villus structures *in vitro* without a mesenchymal niche. Nature 459, 262–265 (2009). 19329995 10.1038/nature07935

[b5] Costello C. M. *et al.* Synthetic small intestinal scaffolds for improved studies of intestinal differentiation. Biotechnol Bioeng 111, 1222–1232 (2014). 24390638 10.1002/bit.25180 PMC4233677

[b6] Kim H. J. & Ingber D. E. Gut-on-a-Chip microenvironment induces human intestinal cells to undergo villus differentiation. Integr Biol (Camb) 5, 1130–1140 (2013). 23817533 10.1039/c3ib40126j

[b7] Deng X., Zhang G., Shen C., Yin J. & Meng Q. Hollow fiber culture accelerates differentiation of Caco-2 cells. Appl Microbiol Biotechnol 97, 6943–6955 (2013). 23689647 10.1007/s00253-013-4975-x

[b8] Fisher M. B. & Mauck R. L. Tissue engineering and regenerative medicine: recent innovations and the transition to translation. Tissue Eng Part B Rev 19, 1–13 (2013). 23253031 10.1089/ten.teb.2012.0723 PMC3564480

[b9] Yu J., Carrier R. L., March J. C. & Griffith L. G. Three dimensional human small intestine models for ADME-Tox studies. Drug Discov Today 19, 1587–1594 (2014). 24853950 10.1016/j.drudis.2014.05.003

[b10] Hilgendorf C. *et al.* Caco-2 versus Caco-2/HT29-MTX co-cultured cell lines: permeabilities via diffusion, inside- and outside-directed carrier-mediated transport. J Pharm Sci 89, 63–75 (2000). 10664539 10.1002/(SICI)1520-6017(200001)89:1<63::AID-JPS7>3.0.CO;2-6

[b11] Kim H. J., Huh D., Hamilton G. & Ingber D. E. Human gut-on-a-chip inhabited by microbial flora that experiences intestinal peristalsis-like motions and flow. Lab Chip 12, 2165–2174 (2012). 22434367 10.1039/c2lc40074j

[b12] Louis P., Hold G. L. & Flint H. J. The gut microbiota, bacterial metabolites and colorectal cancer. Nat Rev Microbiol 12, 661–672 (2014). 25198138 10.1038/nrmicro3344

[b13] Wright W. E. & Shay J. W. Inexpensive low-oxygen incubators. Nat Protoc 1, 2088–2090 (2006). 17487199 10.1038/nprot.2006.374

[b14] Sommer F. & Backhed F. The gut microbiota–masters of host development and physiology. Nat Rev Microbiol 11, 227–238 (2013). 23435359 10.1038/nrmicro2974

[b15] Grant C. N. *et al.* Human and Mouse Tissue-Engineered Small Intestine Both Demonstrate Digestive And Absorptive Function. Am J Physiol Gastrointest Liver Physiol ajpgi 00111 02014 (2015). 10.1152/ajpgi.00111.2014 PMC4398842 25573173

[b16] Watson C. L. *et al.* An *in vivo* model of human small intestine using pluripotent stem cells. Nat Med 20, 1310–1314 (2014). 25326803 10.1038/nm.3737 PMC4408376

[b17] Vepari C. & Kaplan D. L. Silk as a Biomaterial. Prog Polym Sci 32, 991–1007 (2007). 19543442 10.1016/j.progpolymsci.2007.05.013 PMC2699289

[b18] Altman G. H. *et al.* Silk-based biomaterials. Biomaterials 24, 401–416 (2003). 12423595 10.1016/s0142-9612(02)00353-8

[b19] Kim H. J., Kim U. J., Vunjak-Novakovic G., Min B. H. & Kaplan D. L. Influence of macroporous protein scaffolds on bone tissue engineering from bone marrow stem cells. Biomaterials 26, 4442–4452 (2005). 15701373 10.1016/j.biomaterials.2004.11.013

[b20] Tang-Schomer M. D. *et al.* Bioengineered functional brain-like cortical tissue. Proc Natl Acad Sci USA (2014). 10.1073/pnas.1324214111 PMC4183301 25114234

[b21] Mahler G. J., Shuler M. L. & Glahn R. P. Characterization of Caco-2 and HT29-MTX cocultures in an *in vitro* digestion/cell culture model used to predict iron bioavailability. J Nutr Biochem 20, 494–502 (2009). 18715773 10.1016/j.jnutbio.2008.05.006

[b22] Lahar N. *et al.* Intestinal subepithelial myofibroblasts support *in vitro* and *in vivo* growth of human small intestinal epithelium. PLoS One 6, e26898 (2011). 22125602 10.1371/journal.pone.0026898 PMC3219641

[b23] Cheng H. Origin, differentiation and renewal of the four main epithelial cell types in the mouse small intestine. IV. Paneth cells. Am J Anat 141, 521–535 (1974). 4440634 10.1002/aja.1001410406

[b24] He G. *et al.* Noninvasive measurement of anatomic structure and intraluminal oxygenation in the gastrointestinal tract of living mice with spatial and spectral EPR imaging. Proc Natl Acad Sci USA 96, 4586–4591 (1999). 10200306 10.1073/pnas.96.8.4586 PMC16376

[b25] Marra A. & Isberg R. R. Invasin-dependent and invasin-independent pathways for translocation of Yersinia pseudotuberculosis across the Peyer’s patch intestinal epithelium. Infect Immun 65, 3412–3421 (1997). 9234806 10.1128/iai.65.8.3412-3421.1997 PMC175483

[b26] Cecchini G., Schroder I., Gunsalus R. P. & Maklashina E. Succinate dehydrogenase and fumarate reductase from Escherichia coli. Biochim Biophys Acta 1553, 140–157 (2002). 11803023 10.1016/s0005-2728(01)00238-9

[b27] Shu X. *et al.* A genetically encoded tag for correlated light and electron microscopy of intact cells, tissues, and organisms. PLoS Biol 9, e1001041 (2011). 21483721 10.1371/journal.pbio.1001041 PMC3071375

[b28] Bolin I., Portnoy D. A. & Wolf-Watz H. Expression of the temperature-inducible outer membrane proteins of yersiniae. Infect Immun 48, 234–240 (1985). 3980086 10.1128/iai.48.1.234-240.1985 PMC261940

[b29] Crimmins G. T. *et al.* Identification of MrtAB, an ABC transporter specifically required for Yersinia pseudotuberculosis to colonize the mesenteric lymph nodes. PLoS Pathog 8, e1002828 (2012). 22876175 10.1371/journal.ppat.1002828 PMC3410872

[b30] Matz M. V., Lukyanov K. A. & Lukyanov S. A. Family of the green fluorescent protein: journey to the end of the rainbow. Bioessays 24, 953–959 (2002). 12325128 10.1002/bies.10154

[b31] Haycock J. W. 3D cell culture: a review of current approaches and techniques. Methods Mol Biol 695, 1–15 (2011). 21042962 10.1007/978-1-60761-984-0_1

[b32] Linden S. K., Sutton P., Karlsson N. G., Korolik V. & McGuckin M. A. Mucins in the mucosal barrier to infection. Mucosal Immunol 1, 183–197 (2008). 19079178 10.1038/mi.2008.5 PMC7100821

[b33] Lv Q. & Feng Q. Preparation of 3-D regenerated fibroin scaffolds with freeze drying method and freeze drying/foaming technique. J Mater Sci Mater Med 17, 1349–1356 (2006). 17143767 10.1007/s10856-006-0610-z

[b34] Wray L. S. *et al.* A silk-based scaffold platform with tunable architecture for engineering critically-sized tissue constructs. Biomaterials 33, 9214–9224 (2012). 23036961 10.1016/j.biomaterials.2012.09.017 PMC3479404

[b35] Ferruzza S., Rossi C., Scarino M. L. & Sambuy Y. A protocol for *in situ* enzyme assays to assess the differentiation of human intestinal Caco-2 cells. Toxicol In Vitro 26, 1247–1251 (2012). 22123491 10.1016/j.tiv.2011.11.007

[b36] Chen Y. & Gridley T. Compensatory regulation of the Snai1 and Snai2 genes during chondrogenesis. J Bone Miner Res 28, 1412–1421 (2013). 23322385 10.1002/jbmr.1871 PMC3663919

[b37] Lovett M., Rockwood D., Baryshyan A. & Kaplan D. L. Simple modular bioreactors for tissue engineering: a system for characterization of oxygen gradients, human mesenchymal stem cell differentiation, and prevascularization. Tissue Eng Part C Methods 16, 1565–1573 (2010). 20528664 10.1089/ten.tec.2010.0241 PMC2988631

[b38] Jones S. E. & Versalovic J. Probiotic Lactobacillus reuteri biofilms produce antimicrobial and anti-inflammatory factors. BMC Microbiol 9, 35 (2009). 19210794 10.1186/1471-2180-9-35 PMC2653509

[b39] Shu X. *et al.* A genetically encoded tag for correlated light and electron microscopy of intact cells, tissues, and organisms. PLoS Biol 9, e1001041 (2011). 21483721 10.1371/journal.pbio.1001041 PMC3071375

