# Supplementary material for: Robust bioengineered 3D functional human intestinal epithelium
Source: Sci Rep. 2015 Sep 16;5:13708. doi: 10.1038/srep13708 (PMC4571649; doi:10.1038/srep13708)
Supplement: Supplementary Information [file srep13708-s1.doc]

**Supporting Information**

Robust bioengineered 3D functional human intestinal epithelium

Ying Chen^1*^, Yinan Lin^1*^, Kimberly M. Davis^2^, Qianrui Wang^1^, Jelena Rnjak-Kovacina^1,3^, Chunmei Li^1^, Ralph R. Isberg^2^, Carol A. Kumamoto^2^, Joan Mecsas^2^, David L. Kaplan^1^†

^1^Department of Biomedical Engineering, Tufts University, 4 Colby Street, Medford, MA 02155, USA.

^2^Department of Molecular Biology and Microbiology, Tufts University School of Medicine, 145 Harrison Ave, Boston, MA 02111, USA.

^3^Graduate School of Biomedical Engineering, UNSW Australia, Sydney, NSW, Australia.

†Corresponding author. Email: david.kaplan@tufts.edu.

*These authors contributed equally to this work.

*Supporting Information Texts*

*Supporting Information Figures*

*Supporting Information Table*

**Supporting Information Text 1 Tunability of 3D silk scaffolds**

The dimensions of the bulk scaffold and the hollow channels are tunable and the porous scaffolding can be assembled with dimensions that range from millimeters to centimeters by adjusting the size of the PDMS molds. The diameter of the hollow channels and the luminal patterns were determined by the diameter and the surface features of the wires/rods placed in the PDMS molds, thus any channel diameter or luminal pattern is feasible, as we have shown previously with prechanneled systems for vascular-like needs in tissue regeneration [^36^](#_ENREF_36). The 2 mm diameter hollow channels can be increased to match relevant diameters of human intestines (5 cm for large and 2.5 cm for small, on average). The dimensions of the lumens and patterns in our system are adjustable by using different sizes of Teflon-coated wires and Nylon pan head machine screws. Further refinements would be important to assess to further optimize the system. The established low oxygen profiles could be subjected to further changes, such as a result of an increase or decrease in lumen diameter, while conventional engineering-based techniques that fine-tune oxygen supply-demand balances could be employed to address this issue. The length of the scaffold, for example, can be extended and additionally the lumen channel can be dynamically perfused at desired oxygen conditions. In the human body, the small intestine is lined by villi. Previous efforts have been made to recreate the complex villus-like topography of the small intestine for in vitro intestine tissue models [^37-40^](#_ENREF_37). However, the challenge of fabricating villus-like topography on the inner surface of a 3D hollow structure has been limited to flat substrates, which also limit relevance to *in vivo* 3D structures of the small intestine. The tunable scaffold system used here allowed a well-defined threaded pattern, mimicking intestinal villus projections to be integrated into the hollow lumen by using threaded nylon rods during scaffold formation. Compared to non-patterned lumens, the functional surface area of the patterned lumen was significantly increased by these threaded projections resulting in a more physiologically and physically relevant 3D microenvironment for the growth of human intestinal cells. Based on the topographical anatomy of human intestine, villus features are only found in small intestine, therefore, the 3D scaffolds without patterns could be potentially used for large intestine tissues to provide comparable outcomes.

**Supporting Information Text2 Potentiality of 3D silk scaffolds**

In the present study we used the simplest version of the compartmentalized scaffolding, a hollow space within a porous bulk matrix, to engineer intestinal tissues. The tunable 3D system can be further processed for more complex features of the human intestine to enhance function, such as by including microchannels by inserting micro-sized Teflon-coated wires during scaffold formation. These microchannels can be used to include different cell types and immune components, such as endothelial cells, smooth muscle cells, and immune cells (e.g. neutrophils, monocytes, macrophages) for intestine tissue engineering and study. This system could also house human primary large and small intestinal epithelial cells. Furthermore, the 3D scaffolds could be perfused in a bioreactor system to achieve dynamic culture or flow to assess impact on outcomes. In the perfusion system, the epithelial barrier permeability and uptake rate can be evaluated. Overall, the ability to produce a human intestine tissue model in vitro that effectively recapitulates many complex functions of the native human intestine, and remains functional for months, provides a new system with which to study many aspects of human physiology, infections and treatments.

**Supporting Information Figure 1** **Immunostaining of MUC-2 (a marker of goblet cells, green) on Caco-2/HT29-MTXcells seeded on 3D scaffolds for 24 hours**


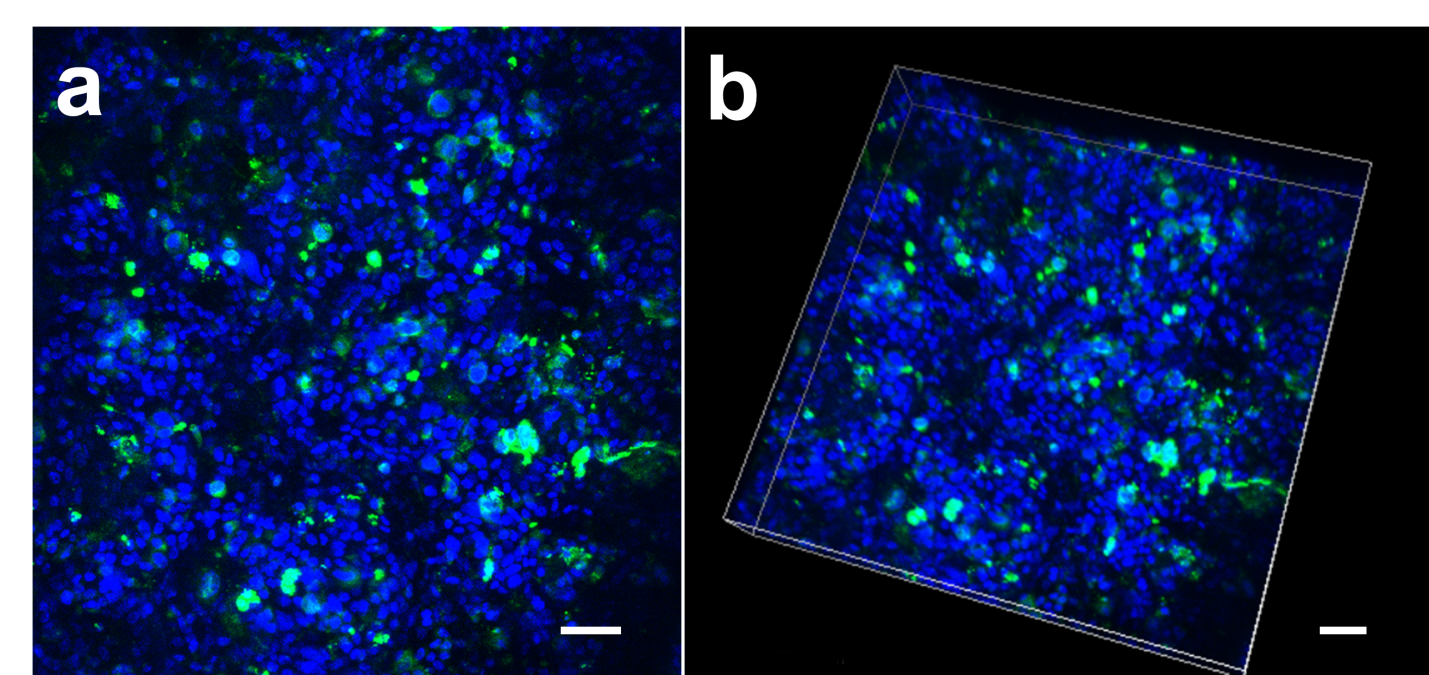


(**a**, **b**) 3D reconstruction of Z-stack images (**a**) and 3D confocal view (**b**) of the immunostaining of MUC-2 (a marker of goblet cells, green) on Caco-2/HT29-MTXcells seeded on 3D scaffolds for 24 hours showed the seeding ratio of Caco-2 and HT29-MTX cells is around 3:1. Scale bar = 50µm.

**Supporting Information Figure 2 Immunostaining of E-cadherin, Villin of Caco-2/HT29-MTX**


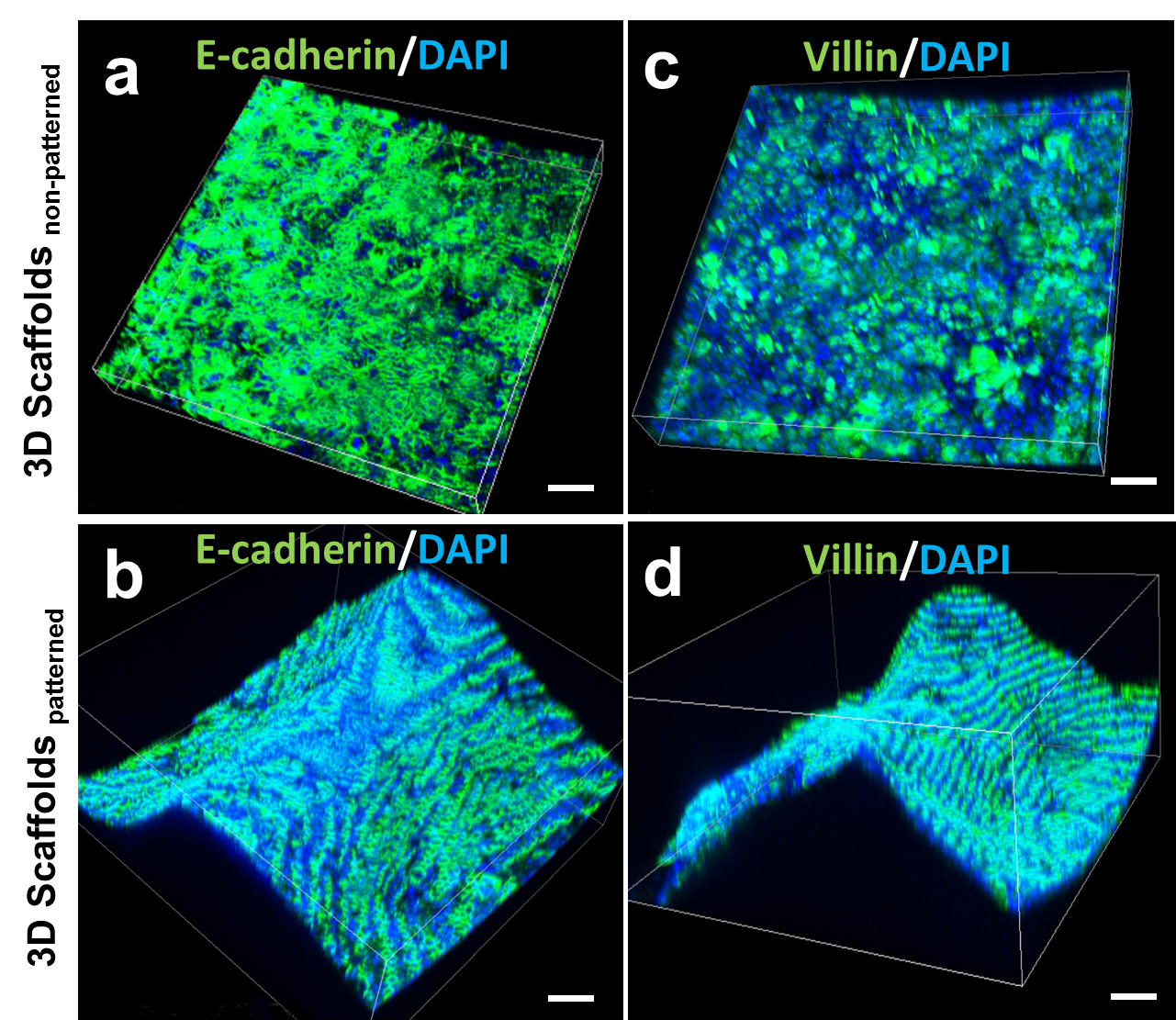


Confocal microscopy images of immunostaining of E-cadherin (**a**,**b**), Villin (**c** ,**d**), Caco-2/HT29-MTX cultured on 2D non-patterned (**a,c**) and patterned (**b,d**) 3D silk scaffolds 21 days post cell seeding. Scale bar = 60µm.

**Supporting Information Figure 3 Cells grown on scaffolds formed columnar structure**


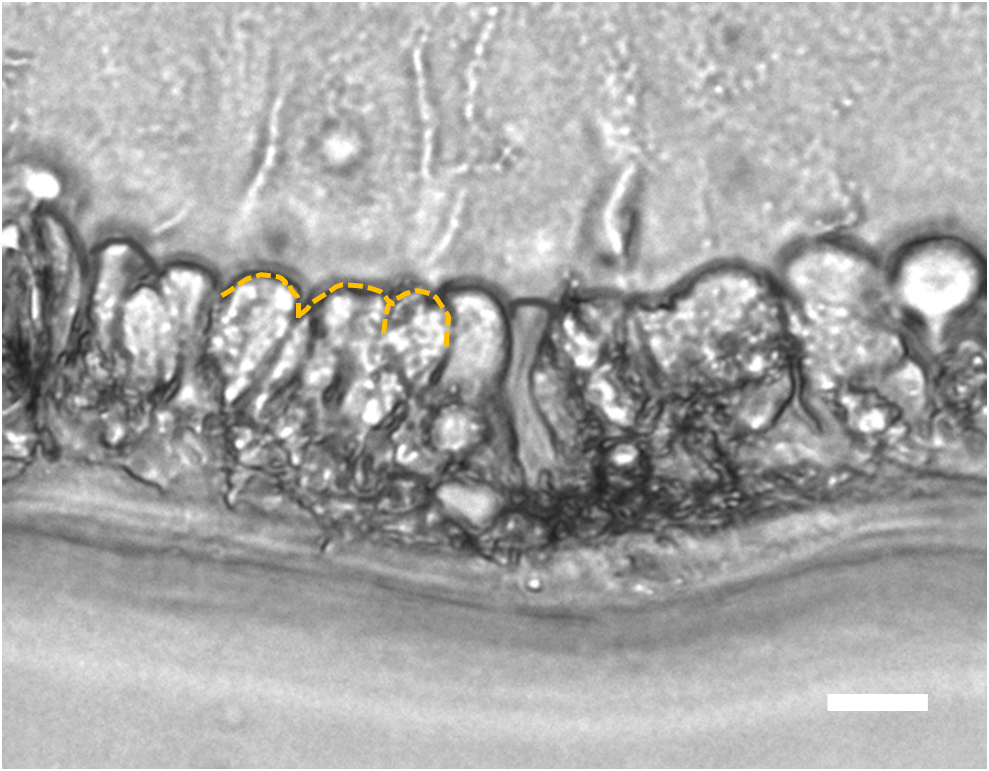


Cells grown on scaffolds formed columnar structure, scale bar, 10 μm.

**Supporting Information Figure 4 Intestinal epithelial cells grown on 3D scaffolds possess higher packing density of microvilli and formed continuous brush borders across the cells earlier compared to cells grown on 2D transwells**


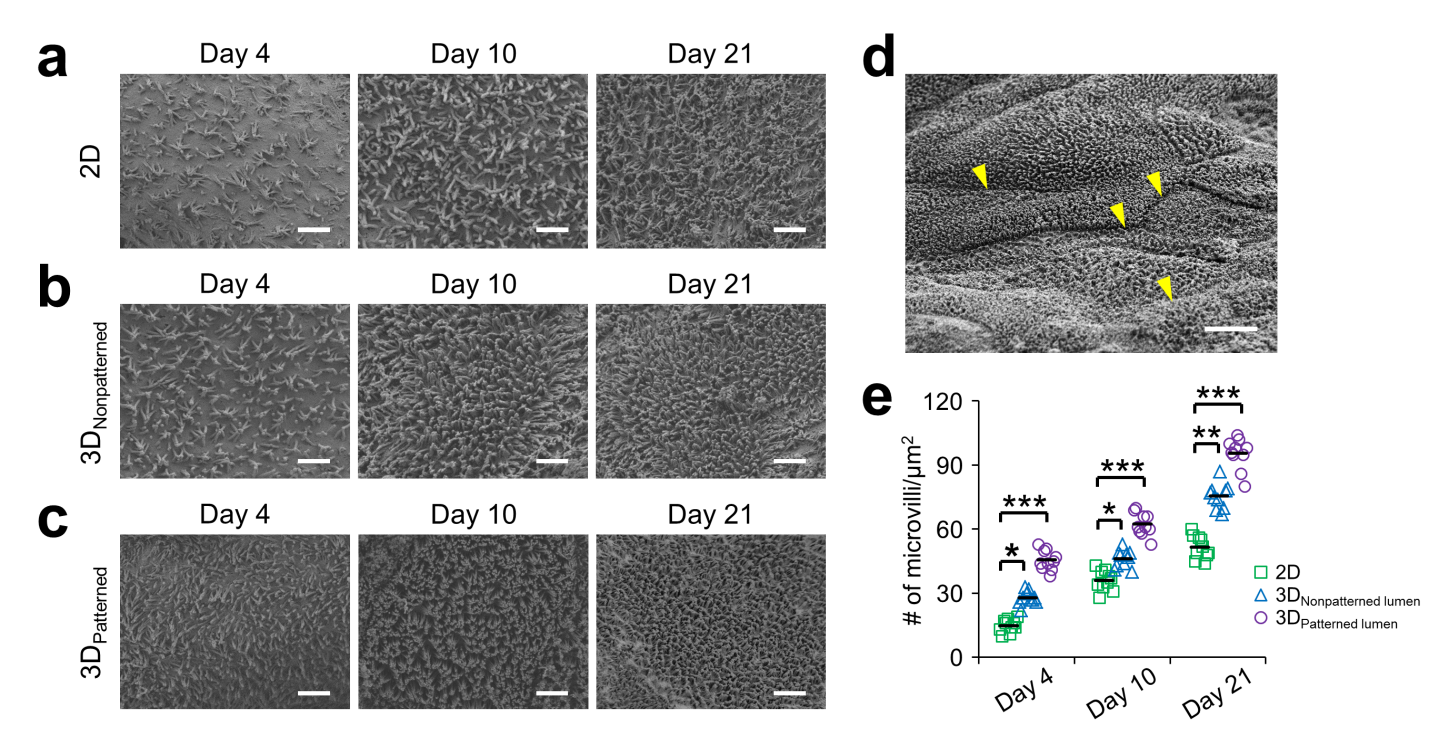


(**a**-**c**) SEM of intestinal epithelial cells grown on 2D transwells (**a**), and non-patterned (**b**) and patterned (**c**) 3D silk scaffolds from a differentiation time series (days 4, 10 and 21). Scale bar = 1µm. (**d**) SEM of intestinal epithelial cells grown on 3D scaffolds from day 10 showed a continuous brush border indicated by yellow arrowheads across the cells. Scale bar = 2µm. (**e**) Quantification of microvilli of epithelial cells grown on 2D and 3D systems. n = 10 in each group, ***p<0.001, **p<0.01, *p<0.05.

**Supporting Information Figure 5** **Live stain of scaffolds cultured with H-InMyoFib cells**


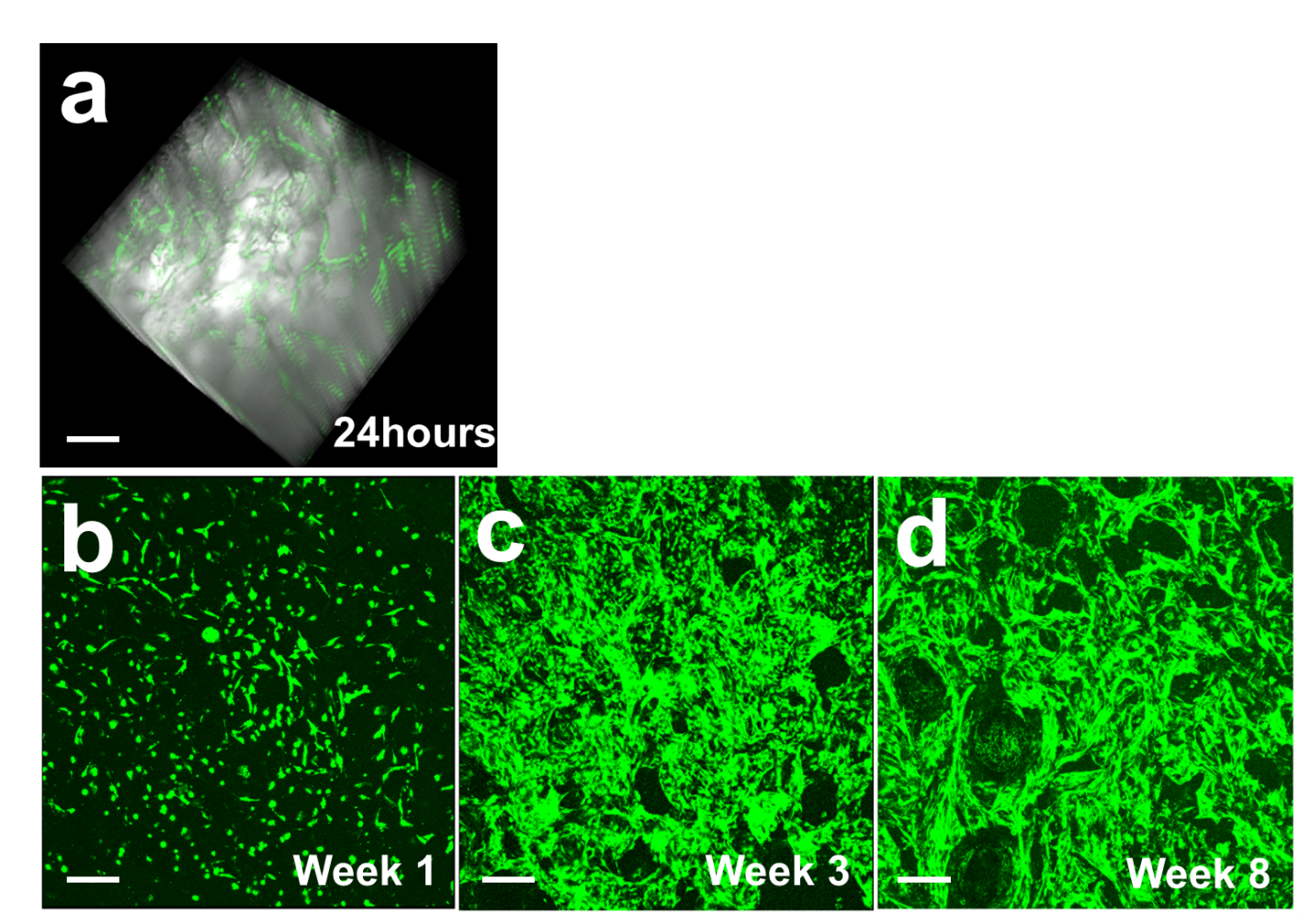


(**a**) Live stain of scaffolds cultured with H-InMyoFib cells after 24 hours of cell seeding indicated that H-InMyoFib cells were successfully delivered into the silk scaffolds. Scale bar = 200µm. (**b**-**d**) Confocal Z stack images of live stain show that H-InMyoFib cells could be cultured on the scaffold bulks for at least 8 weeks. Scale bar = 200µm.

**Supporting Information Figure 6 Quantification of gene expression levels of ZO-1, E-cadherin, and Muc-2 by qRT-PCR**


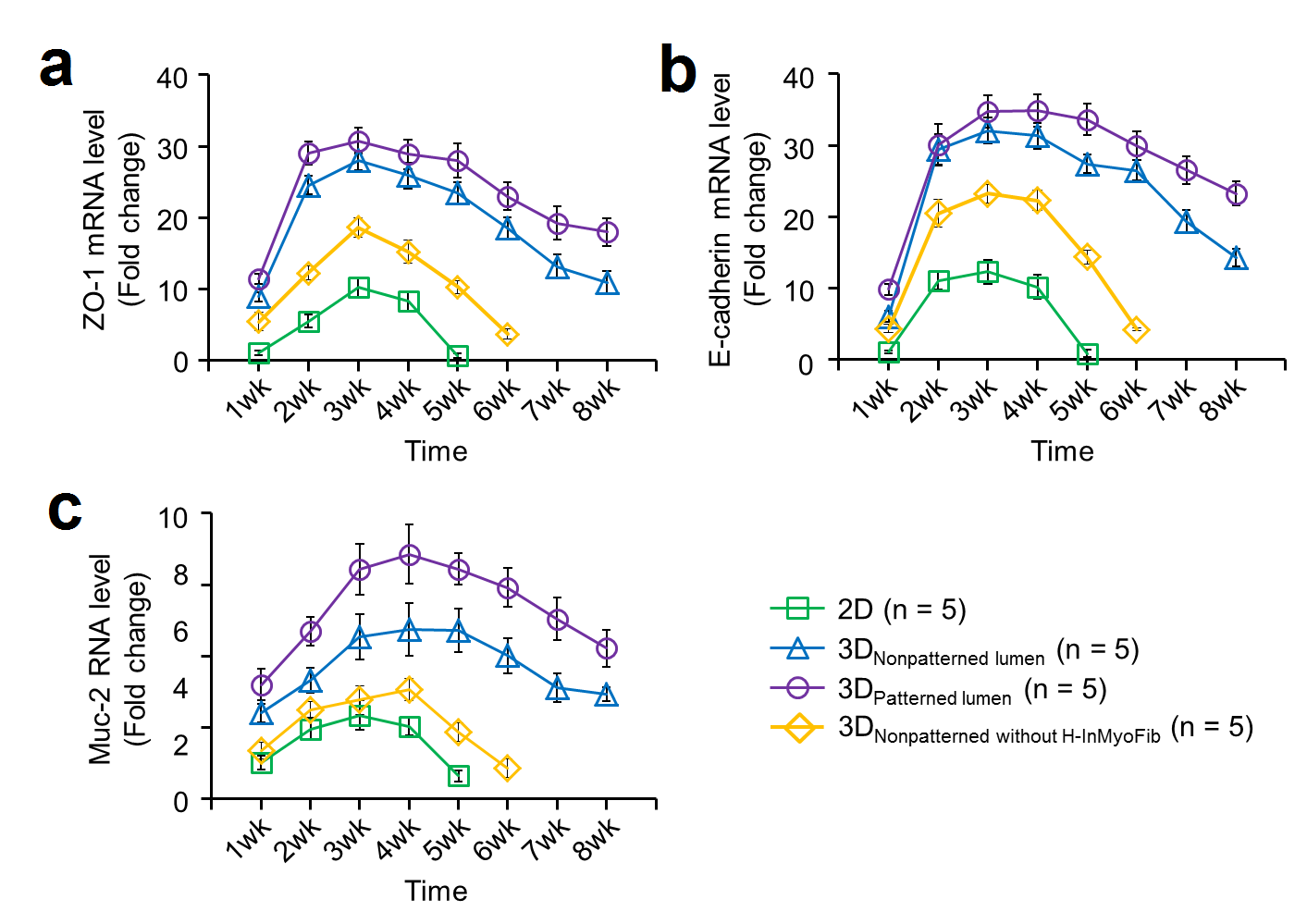


The deletion of H-InMyoFibs in intestinal epithelial cultures in 3D scaffold system produced a shorter-term growth and differentiation of the epithelial cells based on the analysis of epithelial cells grown on 3D non-patterned silk scaffolds without the support of H-InMyoFibs (yellow curve). Gene expression levels of ZO-1(**a**), E-cadherin (**b**), and Muc-2 (**c**), were evaluated by quantitative reverse transcription-polymerase chain reaction (qRT-PCR). Data is presented as mean ± SEM, n=5 in each group, p<0.001.

**Supporting Information Figure 7** **Expression of the *P_frdA_::gfp* and *P_frdA_::miniSOG* constructs.**


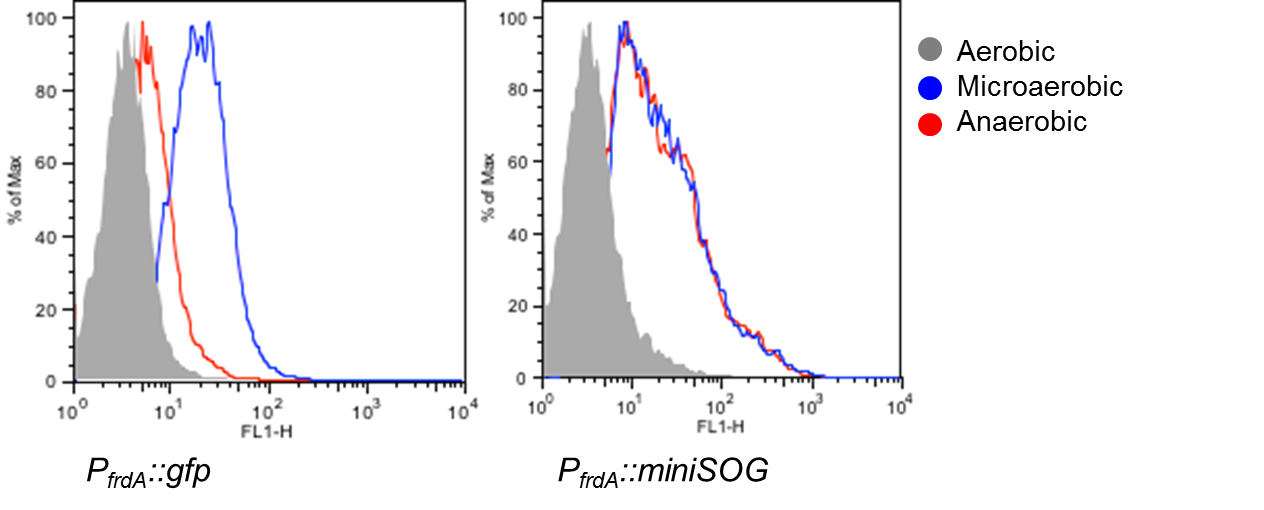


*Y. pseudotuberculosis* expressing *P_frdA_::gfp* or *P_frdA_::miniSOG* were grown aerobically overnight, diluted 1:100 into fresh media, and grown under aerobic, microaerobic, or anaerobic conditions for 2 hours. Cells were pelleted, fixed in 4% paraformaldehyde, and fluorescence was detected by flow cytometry. GFP and miniSOG fluorescence were detected in the FL1 channel, which is shown on the x-axis.

**Supporting Information Figure 8 Immunostaining of ZO-1 and ALP staining on cells grown on scaffolds after eight week post cell seeding**


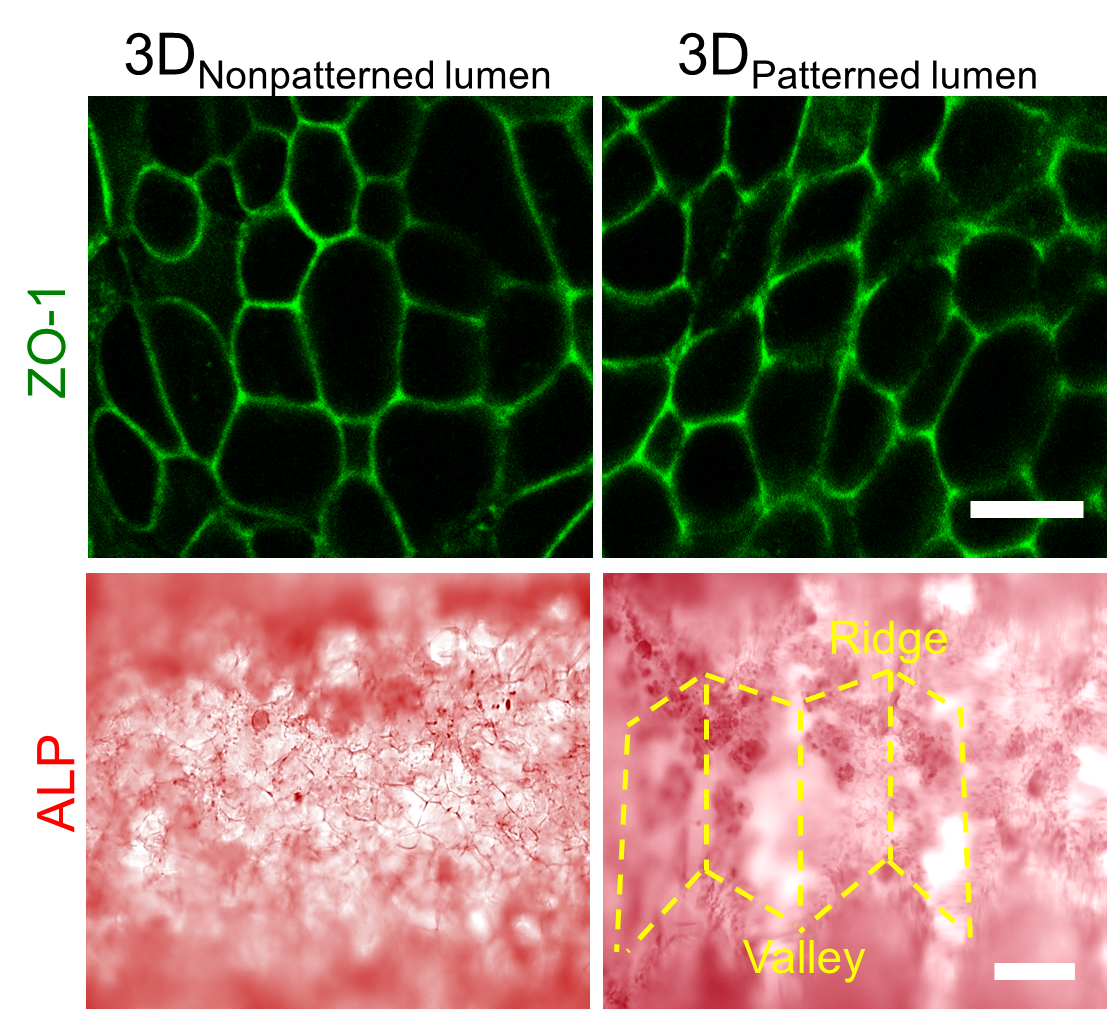


ZO-1 immunostaining (Scale bars, 10 μm) and ALP staining (Scale bars, 150 μm) on the epithelial cells seeded in the scaffold lumens at week 8 post cell seeding demonstrated epithelial cells still maintained cell phenotypes at week 8.

**Supporting Information Figure 9 Lactobacillus rhamnosus GG (LGG) colonization on intestinal epithelial cells**


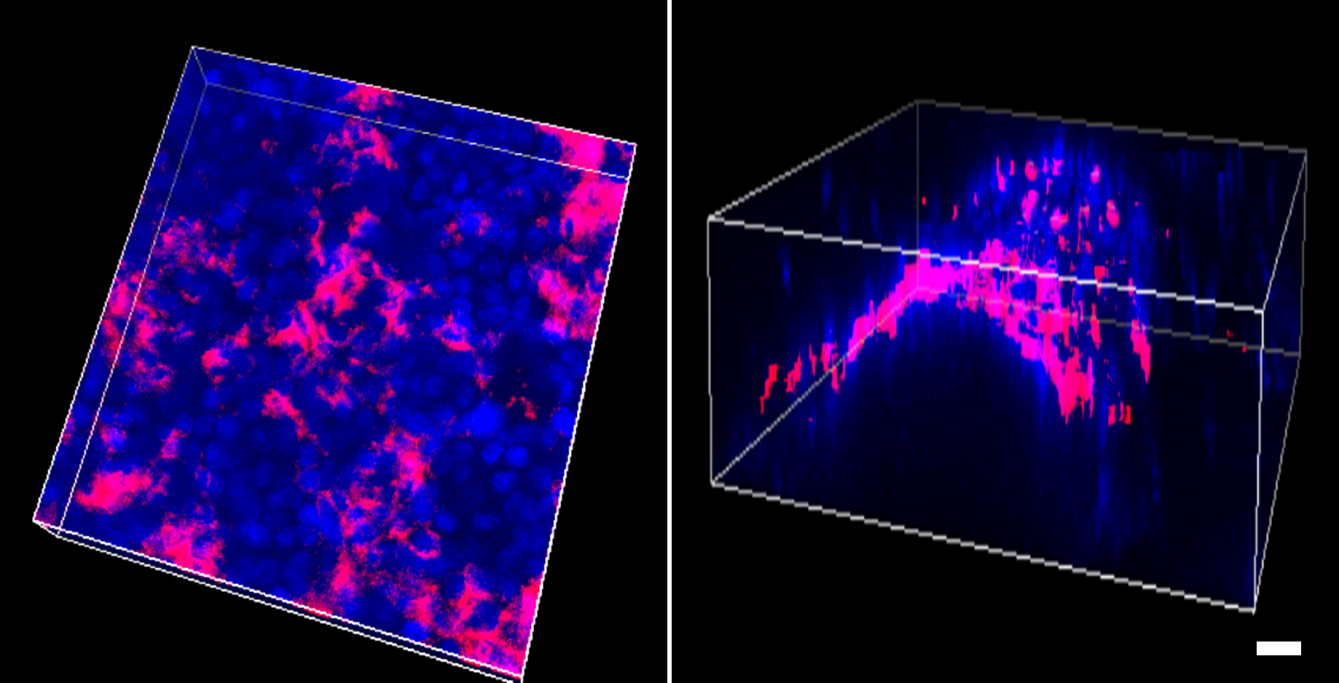


Laser scanning confocal microscopy images of Lactobacillus rhamnosus GG (LGG) colonization after co-cultured with intestinal epithelial cells for 4 hours. The red fluorescence shows live-stained LGG colonies attached to the epithelium grown on non-patterned (left) and patterned (right) 3D scaffolds. The blue fluorescence shows the nuclei of the epithelial cells. Scale bar = 25µm.

**Supporting Information Table 1 qRT‐PCR primer list**

| **Gene products** | Forward | Reverse |
| --- | --- | --- |
| GAPDH | GAAGGTGAAGGTCGGAGTC | GAAGATGGTGATGGGATTTC |
| ZO-1 | CTGGTGAAATCCCGGAAAAATGA | TTGCTGCCAAACTATCTTGTGA |
| E-caherin | ATCGGTTGTTCAATGCGTCC | CCTTCAGGATTTGGTACATGACA |
| Villin | CGGAAAGCACCCGTATGGAG | CGTCCACCACGCCTACATAG |
| SI | TCCAGCTACTACTCGTGTGAC | CCCTCTGTTGGGAATTGTTCTG |
| *frdA* promoter (*gfp*) | GCAGCATGCGGTGGTTAACTCCGA | TCTCCTTTACTCATTGCGCTTCTCCATT |
| *gfp* | AATGGAGAAGCGCAATGAGTAAAGGAGA | GGAGGATCCTTTGTATAGTTCATCCATGCC |
| *frdA* promoter (*miniSOG*) | GCAGCATGCGGTGGTTAACTCCGA | AAACTTTTCTCCATTGCGCTTCTCCAT |
| *miniSOG* | ATGGAGAAGCGCAATGGAGAAAAGTTT | GGAGGATCCTTATCCATCCAGCTGCAC |
